# Key Role of Polyphosphoinositides in Dynamics of Fusogenic Nuclear Membrane Vesicles

**DOI:** 10.1371/journal.pone.0023859

**Published:** 2011-09-08

**Authors:** Vanessa Zhendre, Axelle Grélard, Marie Garnier-LHomme, Sébastien Buchoux, Banafshé Larijani, Erick J. Dufourc

**Affiliations:** 1 Chemistry and Biology of Membranes and Nanoobjects (CBMN), UMR5248 - CNRS-Université Bordeaux-Institut Polytechnique de Bordeaux, Pessac, France; 2 Cell Biophysics Laboratory, Lincoln's Inn Fields Laboratories, Cancer Research UK, London, United Kingdom; 3 UMR 6022 - Génie Enzymatique et Cellulaire, Université Picardie Jules Verne (UPJV), Amiens, France; The University of Kansas Medical Center, United States of America

## Abstract

The role of phosphoinositides has been thoroughly described in many signalling and membrane trafficking events but their function as modulators of membrane structure and dynamics in membrane fusion has not been investigated. We have reconstructed models that mimic the composition of nuclear envelope precursor membranes with naturally elevated amounts of phosphoinositides. These fusogenic membranes (membrane vesicle 1(MV1) and nuclear envelope remnants (NER) are critical for the assembly of the nuclear envelope. Phospholipids, cholesterol, and polyphosphoinositides, with polyunsaturated fatty acid chains that were identified in the natural nuclear membranes by lipid mass spectrometry, have been used to reconstruct complex model membranes mimicking nuclear envelope precursor membranes. Structural and dynamic events occurring in the membrane core and at the membrane surface were monitored by solid-state deuterium and phosphorus NMR. “MV1-like” (PC∶PI∶PIP∶PIP_2_, 30∶20∶18∶12, mol%) membranes that exhibited high levels of PtdIns, PtdInsP and PtdInsP_2_ had an unusually fluid membrane core (up to 20% increase, compared to membranes with low amounts of phosphoinositides to mimic the endoplasmic reticulum). “NER-like” (PC∶CH∶PI∶PIP∶PIP_2_, 28∶42∶16∶7∶7, mol%) membranes containing high amounts of both cholesterol and phosphoinositides exhibited liquid-ordered phase properties, but with markedly lower rigidity (10–15% decrease). Phosphoinositides are the first lipids reported to counterbalance the ordering effect of cholesterol. At the membrane surface, phosphoinositides control the orientation dynamics of other lipids in the model membranes, while remaining unchanged themselves. This is an important finding as it provides unprecedented mechanistic insight into the role of phosphoinositides in membrane dynamics. Biological implications of our findings and a model describing the roles of fusogenic membrane vesicles are proposed.

## Introduction

Membrane fusion is required for membrane trafficking, regeneration of various sub-cellular compartments after cell division, and cell growth. It is a process that is regulated by both proteins and lipids. Until recently the molecular mechanisms of membrane fusion were thought to be driven mainly by Rab GTPases and SNARE proteins. It is only in the past few years that researchers have reconstructed the accepted models by studying the involvement of phosphoinositides and their derivatives such as diacylglycerol [1], [2], [3], [4].

These phospholipids were mainly recognised as second messengers and their effect on membrane dynamics and structure was not correlated with their role as signalling molecules. The combined *in vitro* and *in vivo* research on the participation of phosphoinositides in the regulation of membrane fusion has resulted in a re-evaluation of the “SNARE model” to include the higher phosphorylated phosphoinositides [5], [6]. One particular example of membrane fusion is the regulation of nuclear envelope assembly. The nuclear envelope is disassembled and reassembled at each mitosis in typical animal cells. The processes of disassembly and reassembly may also occur at interphase, in a coordinated fashion, in nuclei sharing a common cytoplasm, for example in fertilised eggs. Male nuclear envelopes however are disassembled and reassembled in all cases. The study of male pronuclear membrane formation in fertilised sea urchin oocytes, using a cell free assay, has revealed several novel features, especially regarding the role of phospholipids during nuclear membrane formation [7], [8]. The lipidome of these nuclear envelope precursor membranes has been analysed with High Performance Liquid Chromatography-Electrospray Ionisation Tandem Mass Spectrometry (HPLC-ESI-MS/MS) and shown that it is rich in unsaturated polyphosphoinositides, including the PLCγ substrate PtdIns(4,5)P_2_. The non-endoplasmic reticulum derived vesicles (MV1) are located in the cortex of the oocyte and the nuclear envelope remnants (NER) are conserved membrane regions on the acrosomal and centriolar fossae of the sperm nucleus. These membranous compartments are crucial in the assembly of the male pronucleus envelope. Both of these membranes are enriched in polyphosphoinositides [5], [7], [9], with NERs containing high levels of cholesterol. The endoplasmic reticulum-derived vesicles (MV2) form the bulk of the nuclear envelope and have a typical phosphoinositide composition.

In the regulation of membrane fusion the association of highly phosphorylated phosphoinositides with SNAREs was observed, but only speculations on how fusion may be regulated by the polyphosphoinositides were proposed [5]. To study the implications of highly phosphorylated phosphoinositides in membrane dynamics, complex model membranes with similar lipid compositions to MV1, MV2 and NERs were constructed using PtdCho, PtdEth, PtdSer, Cholesterol, PtdIns, PtdInsP and PtdInsP_2_ lipids. Head group and chain composition were matched as closely as possible with the composition of natural precursor membranes analysed by HPLC-ESI-MS/MS [7], [9]. Their structure and dynamics were studied by ^31^P and ^2^H solid-state NMR spectroscopy, an ideal non-invasive, non-destructive and quantitative methodology for probing membrane fluidity [10], [11], [12].

We have found for the first time that “MV1-like” membranes are disordered membranes and the effect of highly phosphorylated phosphoinositides was to render the membrane more fluid. Moreover, phosphoinositide lipids increased the fluidity of “NER-like” membranes that would otherwise be rigid in the presence of high levels of cholesterol. We also observed, phosphoinositides control the orientation dynamics of other lipids in these membranes, while remaining unchanged themselves. These effects have considerable biological implications in the understanding of the mechanisms of membrane fusion.

## Materials and Methods

### Materials

Lipids were purchased from Avanti Polar Lipids, Inc. (Alabaster, Alabama, USA): 1-palmitoyl(^2^H_31_)-2-oleoyl-*sn*-glycero-3-phosphocholine (POPC-^2^H_31_), L-α-phosphatidylinositol (Liver, Bovine: PtdIns; mainly composed of {18∶0/20∶4}), L-α-phosphatidylinositol-4-phosphate (Brain, Porcine: PtdInsP; mainly composed of {18∶0/20∶4}), L-α-phosphatidylinositol-4,5-bisphosphate (Brain, Porcine: PtdInsP_2_; mainly composed of {18∶0/20∶4}), 1-palmitoyl-2-oleoyl-*sn*-glycero-3-phosphoethanolamine (POPE), 1-palmitoyl-2-oleoyl-*sn*-glycero-3-phospho-L-serine (POPS) and cholesterol were supplied by Avanti Polar lipids, Inc. (Alabama, USA). All starting materials were used without further purification. All solvents were purchased from VWR International (France), depleted water from Eurisotop (St-Aubin, France), and 4-mm (50 µl) ZrO_2_ rotors and caps from Cortec (Paris, France).

### Preparation of aqueous lipid dispersions

Multilamellar vesicles of lipids (MLV) were made following published procedures [12]. The dry individual lipid powder (1–5 mg) was dispersed at room temperature in 20–100 µl of MOPS buffer (100 Mm KCl, 1 mM MOPS) [13], [14], mixed in a vortex shaker for 5 min, cooled until frozen in liquid nitrogen and warmed up to 30°C. This freeze-thaw cycle was repeated until a homogeneous dispersion was obtained. In the case of mixed systems, lipids were first dissolved in chloroform or chloroform/methanol and subsequently mixed. The solvent was removed by speed vacuum, the residue hydrated with excess water, homogenised and lyophilized once. The resulting “fluffy” powder was hydrated to 95% (w/w), homogenised as described above and placed into a 50- µL ZrO_2_ rotor. Table 1 summarises the molar composition of all the lipid systems used. Depending on the total lipid mass (5 mg) and the ratio of the different lipids, the amount of deuterated lipid ranged from 1 to 3 mg.

**Table 1:** Lipid composition of nuclear membranes [7], [9].

Biological membranes					
	Lipid composition (mol %)			chain composition	
Lipids	MV1	MV2	NERs	MV1	NERs
PtdCho	32.2±4.1	32.8±4.2	17.7±2.1	nd	18∶0/22∶6; 18∶0/22∶5; 16∶0/20∶4
Chol	12	22	42.0±10.0	nd	nd
PtdEth	2.4±1.5	24.7±4.4	6.7±0.4	nd	18∶1/20∶4
PtdAc	1.7±0.5	0	0.6±0.1	nd	14∶0/18∶2
PtdSer	1.9±0.3	4.6±0.2	2.4±0.2	nd	18∶0/22∶5
PtdGly	0	0	1.5±0.1	nd	nd
PtdIns	20.0±3.3	20.1±2.3	10.4±2.5	18∶0/22∶6aa	18∶0/22∶6aa
PtdInsP	18.3±5.1	0.9±0.3	6.8±2.0	18∶0/22∶6aa	18∶0/22∶6aa
PtdInsP_2_	12.0±5.9	0.5±0.4	6.8±0.9	18∶0/22∶6aa	18∶0/22∶6aa
PtdInsP_3_	7.8±3.4	1.6±0.8	5.0±1.3	18∶0/22∶6aa	18∶0/22∶6aa

Phospholipid composition was determined by HPLC-ESI-MS/MS. Cholesterol/cholesteryl esters were determined by colorimetry. Data are expressed as mean±SEM (n = 3 for NER analysis). Sterol content for MV1 and MV2 is representative of two sets of experiments. nd: not determined. The fatty acid chains of MV1 and NER membranes (the diacyl as well as alkylacyl) are polyunsaturated (aa) chains.

### Cholesterol/Cholesteryl Ester (Sterols) content in natural membrane precursors

Cholesterol and cholesteryl ester concentrations were determined from lipid-extracted samples. Lipid pellets resuspended in 5 µl TN buffer and 5 µl of cholesterol standard solutions ranging from 0.3 to 7.8 mM were supplemented with 500 µl of cholesterol liquid stable reagent (Thermo Electron Corporation). The solutions were probe-sonicated (Soniprep 150) at power 10 for 3 seconds and incubated for 5 minutes at 37°C. Absorption was measured at 500 nm. From these assays the relative amounts of cholesterol to total lipids was determined. In Table 1, mol% refers to relative amount of cholesterol compared to total lipids including cholesterol.

### Quantification of phospholipids and fatty acid chains by HPLC coupled to tandem mass spectrometry (HPLC-ESI-MS/MS)

Biological samples were extracted in silanised glassware according to a modified Folch procedure [15]. The extracted samples were centrifuged at 800×g for 15 min at 4°C. The organic phase was dried at 37°C under nitrogen. The lipid pellet was resuspended in 100 µl chloroform/methanol/water (5∶5∶1) and transferred to a 150 µl silanised insert. The lipids were dried under nitrogen and supplemented with 2 µg of phospholipid internal standards. Before use, lipids were resuspended in chloroform/methanol/water (90∶9.5∶0.5) [16]. Mass spectrometry lipid analysis was carried out on an API 3000 instrument equipped with an ESI source (Sciex/Applied Biosystems). Lipids were separated by HPLC prior to detection using a normal phase Luna silica (2) 3 µm column (Phenomenex). Further details have been published previously [16].

### NMR spectroscopy

NMR experiments were carried out using Bruker Avance 400- (9.36 T), 500- (11.75 T) or 700 (16.45 T). ^31^P NMR spectra were acquired at 162 MHz and 283 MHz, using a phase-cycled Hahn-echo pulse sequence with gated broadband proton decoupling [17]. ^2^H NMR experiments on deuterated lipids were performed at 76 MHz and 107 MHz by means of a phase-cycled quadrupolar echo pulse sequence [18]. Typical acquisition parameters were as follows: spectral window of 75–150 kHz for ^31^P NMR, 500 kHz for ^2^H NMR; π/2 pulse widths ranged from 5.5–12.2 µs for ^31^P, and from 2.75–3.50 µs for ^2^H; interpulse delays were of 30–50 µs for ^31^P and 30–40 µs for ^2^H. A recycle delay of 5 s was used for ^31^P and of 1.5 s for ^2^H. Depending on the samples, either 5 k scans were recorded for phosphorous spectra, or 10–100 k scans for deuterium spectra. Quadrature detection was used in all cases. Samples were allowed to equilibrate for at least 30 minutes at a specific temperature. The temperature variation was +/−°C before the acquisition of time dependent signals. Before Fourier transformations a line broadening of 50–300 Hz was applied to the spectra. Phosphorous chemical shifts were obtained relative to 85% H_3_PO_4_ (0 ppm). The reference for solid-state deuterium powder patterns was set to zero and the position of the carrier placed in the middle of the symmetric Pake pattern (powder spectrum).

### Data analysis

Solid-state NMR spectra of MLV are rather complex to analyse because the sample is not aligned with respect to the magnetic field. The spectrum reports on all possible chains or head group orientations and dynamics. Such spectra are known as “powder”- spectra and describe the random orientation of all molecules in the liposome, with respect to the magnetic field. Moreover, since we have used a deuterated palmitic chain the global deuterium spectrum has an additional complexity that results from the superposition of sub-spectra derived from the chemically and dynamically non-equivalent and non-similarly oriented deuterons of the palmitic acyl chain.

For phosphorus spectra also, there may be a superposition due to the presence of several phosphates. In the case of the phosphoinositides, these phosphates may be dynamically non-equivalent and not oriented in the same manner with respect to the membrane surface. Therefore a more quantitative description of the bilayer core and surface orientation and dynamics was obtained using several calculation tools. We used two approaches: one intended to “suppress” the “powder” NMR line shape to obtain sharp lines for more accurate measurements [19]. This procedure is called deconvolution or de-Pakeing and produces simpler oriented-like spectra [20], [21]. The other procedure simulates the entire experimental spectrum accounting for the powder distribution, liposome deformation, different orientation and dynamics of chain segments and the head group phosphates.

Deuterium and phosphorous NMR take advantage of two different magnetic properties, quadrupolar interaction, Δν_Q_, and chemical shielding, Δσ, both of them globally sense for changes in the electric environment of the reporter nucleus. Consequently, complex non-oriented spectra could be simulated by the input of initial predictions of Δν_Q_, Δσ, line widths and relative molecular weights based on parameters from experimental spectra (Dufourc, FORTRAN routines [22], and Buchoux, unpublished). Slight magnetic field deformation of initially spherical MLVs may appear and these were also implemented in the spectral simulations. For long acyl chains like the palmitic chain of POPC, the average orientation in lamellar fluid phases (liquid-disordered or liquid-ordered) is normal to the bilayer surface and peaks in deuterium de-Paked spectra can be attributed to individual C_k_-^2^H bonds [23], [24]. The corresponding bond order parameters, 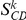, can be calculated from the measurement of individual quadrupolar splittings, 
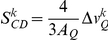
, in between the 90° orientations in powder patterns: 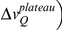. A_Q_ is the static quadrupolar coupling constant (167 kHz, [25]). Often the plateau of quadrupolar splittings is measured, corresponding to labelled positions k = 2 to 8–10. Measurements of the terminal C_16_-^2^H_3_ order parameter can also be performed to report on the dynamics at the centre of the bilayer.

## Results

The study of the structure and dynamics of membranes with high levels of phosphoinositides was performed by making model membrane vesicles with a similar lipid composition to MV1 (PtdCho, PtdIns, PtdInsP, PtdInsP_2_), MV2 (PtdCho, PtdEth, PtdSer, PtdIns, Chol), and NER (PtdCho, Chol, PtdIns, PtdInsP, PtdInsP_2_) membranes. The fatty acyl chain composition of natural MV1, MV2 and NER were determined by using lipid mass spectrometry as described in the Materials and Methods, Table 1. Chains of 18 and 22 carbons were detected with up to 6 double bonds. The model membrane compositions were made as close as possible to natural precursor membranes, Table 2. Control samples with PtdCho plus individual phosphoinositides and/or cholesterol were also measured using both ^2^H- and ^31^P NMR.

**Table 2:** MV1, MV2 and NER-like model membranes.

Model membranes													
		MV1-family						MV2	NER-family				
	Lipid chains	+PI	+PIP	+PIP_2_	+PI +PIP	+PI +PIP_2_	MV1		+Chol	+Chol +PI	+Chol +PI+PIP	+Chol +PI+PIP_2_	NER
POPC	16∶0;18∶1	30	30	30	30	30	30	30	58	28	28	28	28
Chol								20	42	42	42	42	42
POPE	16∶0;18∶1							25					
POPS	16∶0;18∶1							5					
PI^a^	18∶0;20∶4^b^	20			20	20	20	20		30	23	23	16
PIP^a^	18∶0;20∶4^b^		18		18		18				7		7
PIP_2_ a	18∶0;20∶4^b^			12		12	12					7	7

Twelve lipid compositions were prepared using commercially available lipids.

a : PI = PtdIns; PIP = PtdIn**s**P, PIP_2_ = PtdInsP_2_ were obtained from natural membrane (Liver and brain).

b : 18∶0/20∶4 is the dominant fatty acid chain in the chain distribution. Proportions are indicated in mol%, accuracy is 1%. Membrane hydration (mass of lipids/mass of lipids + water) was 95% in all cases.

### Membrane dynamics of “MV1-like” membranes

**Figure 1:**
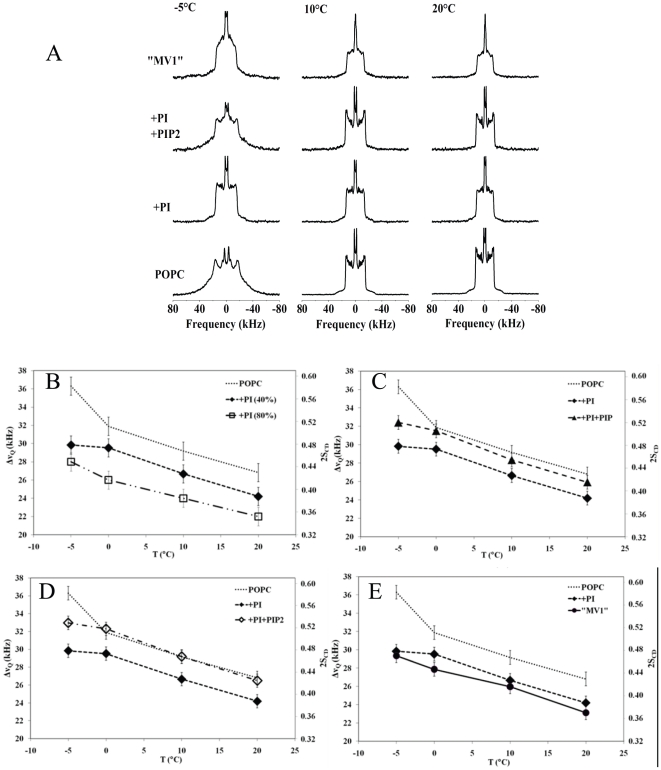
Core fluidity of ptdcho/ptdins model membranes by deuterium wide-line nmr spectroscopy. A-Representative deuterium wide-line NMR spectra of POPC-^2^H_31_ in the absence (bottom) or presence of different PtdIns. The molar ratios are representative of the lipid composition found in MV1: POPC/PtdIns (30/20), POPC/PtdIns/PtdInsP_2_ (30/20/12), and MV1-like: POPC/PtdIns/PtdInsP/PtdInsP_2_ (30/20/18/12). Temperatures are indicated on spectra. Sample hydration (water mass/water + lipid mass) is 95%. Depending on POPC amounts (1–3 mg), each spectrum is the result of 10 k to 80 k cumulative scans. A Lorentzian filtering (LB) of 200–300 Hz was applied prior to Fourier transformation. **FIG. 1B–D** Ordering of chain segments close to glycerol backbone, MV1-like membranes. Thermal variation of the *plateau* (k = 2 to 8–10) quadrupolar splittings of POPC, POPC/PtdIns (30/20 and 10/40) panel B; POPC/PtdIns/PtdInsP (30/20/18), panel C); POPC/PtdIns/PtdInsP_2_ (30/20/12), panel D; and MV1-like model membranes POPC/PtdIns/PtdInsP/PtdInsP_2_ (30/20/18/12), panel E. For comparison, data for pure POPC and POPC/PtdIns (30/20) membranes was added to the graph for the three last compositions. Accuracy of the measure is ±1 kHz. On the double Y-axis the corresponding Carbon-Deuterium order parameter (S_CD_) is shown. Because the average orientation of all *plateau* C–D bonds is at 90° with respect to the long lipid axis, 2 |S_CD_| is plotted to express residual ordering information relative to the bilayer normal.

Figure 1A shows selected spectra of model membranes of various phosphoinositide compositions. Deuterated POPC was chosen as the probe to study a physiologically relevant system [16], [26]. In order to explore different temperature ranges, scans were carried out from −10°C to +40°C. The bottom panel shows selected control POPC-^2^H_31_ spectra. Deuterium solid-state NMR spectra obtained for lipid membranes are complex because they depend on the nature of the lipid phase (lamellar, hexagonal, cubic and micelle), its associated dynamics (molecular order, rigidity and fluidity) and on the number of non-equivalent atoms responsible for the signal [10], [11], [12]. All spectra shown in Figure 1 contain deuterated POPC as a lipid reporter. This leads to the superimposition of at least 15 signals, bearing in mind that the deuterons on the methyl end are equivalent. Although increasing the number of deuterons ensures a greater sensitivity, not all signals can always be resolved. However, the overall spectral shape has been shown to accurately report membrane dynamics. As a rule of thumb the wider the overall shape, the more restricted is lipid dynamics in the membrane [10], [11], [12] In Figure 1A, at −5°C, a superposition of a very wide spectrum (120 kHz width) and a much narrower one is observed. The wide spectrum is axially asymmetric and is characteristic of a L_β′_ (solid ordered, *so*) gel phase. The narrower spectrum is typical of a fluid phase L_a_ (liquid disordered, *ld*). Such coexistence was particularly observed in the middle of the total spectrum where two quadrupolar doublets were clearly identified, one representative of each lipid phase, in almost equivalent amounts. This is indicative of a phase transition occurring at T_m_≈−5°C. At 10°C and 20°C a much narrower axially symmetric spectrum was observed indicative of a single fluid phase. The fine structure of several doublets indicates the specific ordering for each of the lipid chain segments [11].

The decrease in the spectral width indicated the appearance of molecular and intramolecular disorder (molecular anisotropic diffusion and trans-gauche isomerisation). In the lipid mixture of POPC and PtdIns, at 30/20 ratio (40 mol% PtdIns), the spectral width at all three temperatures was reduced. At −5°C, the spectrum did not indicate the presence of a *so* phase, suggesting that T_m_ for this system was lower than −5°C. This result was in agreement with the disordering effect of PtdIns already reported for higher amounts (80 mol%) of PtdIns [27]. To observe the dynamics of membranes with high levels of PtdInsP_2_, model membranes with POPC/PtdIns/PtdInsP_2_ were made at a ratio of 30/20/12. The spectra we measured were very similar to those obtained for pure POPC, suggesting that PtdInsP_2_ counterbalanced the disordering effect of PtdIns. To mimic MV1, the spectra of a model membrane composed of (POPC/PtdIns/PtdInsP/PtdInsP_2_∶30∶20∶18∶12 mol%) were acquired. At three different temperatures the spectra were narrower than pure POPC, especially at −5°C where the gel phase was not detected. The effect of PtdInsP was also acquired but, for clarity, not shown in Figure 1A. The order parameter analysis is reported in Figure 1B–E.

To quantify more accurately these different effects, the plateau of quadrupolar splitting (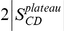 was measured from de-Paked spectra (See Methods) and plotted as a function of temperature (Figure 1B–E). To “translate” quadrupolar splitting into chain ordering the quantity 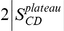 was plotted on a double-y axis. The “plateau” doublet represents the 2–10 carbon chain positions, which are dynamically equivalent, and reports precisely on membrane microfluidity. The factor 2 in the second term accounts for the fact that the average orientation of all plateau carbon-deuterium bonds is at 90° with respect to the long lipid axis. This definition allows us to compare completely disordered (fluid) systems (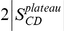 = 0) to fully ordered (rigid) systems (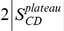 = 1) [28].

Panel 1B reports the effect of different levels of PtdIns on POPC. A highly elevated amount of PtdIns (80 mol%) induced molecular disorder as shown by [27]. The lower amount used here (40 mol%) produced an intermediate effect suggesting that the disordering effect of PtdIns is dose dependent. In Figures 1C and 1D, addition of PtdInsP or PtdInsP_2_ illustrates that order parameters were almost identical to pure POPC, *i.e.*, rendering negligible the PtdIns disordering effect. Figure 1E demonstrates the order parameter for “MV1-like” membranes where all three polyphosphorylated phosphoinositides were mixed with POPC. In this case the membrane was as disordered as for simple POPC/PtdIns, but from the above panels, it was observed that the final effect was the result of the intricate effects of all polyphosphoinositides. By taking the de-Paked spectra into consideration (not shown) the behaviour of the plateau positions (near the glycerol backbone) can be extended to all chain positions (Figure S1).

As a control, MV2 models were also monitored by ^2^H NMR. They behaved similar to POPC/Chol (20 mol%) at 10°C and above. At 0°C and −5°C spectra were characteristic of biphasic gel and fluid systems (Figure S2). Order parameters were extracted from de-Paked spectra and shown for comparison with MV1 and “NER–like” membranes.

### Membrane dynamics of “NER-like” membranes

**Figure 2:**
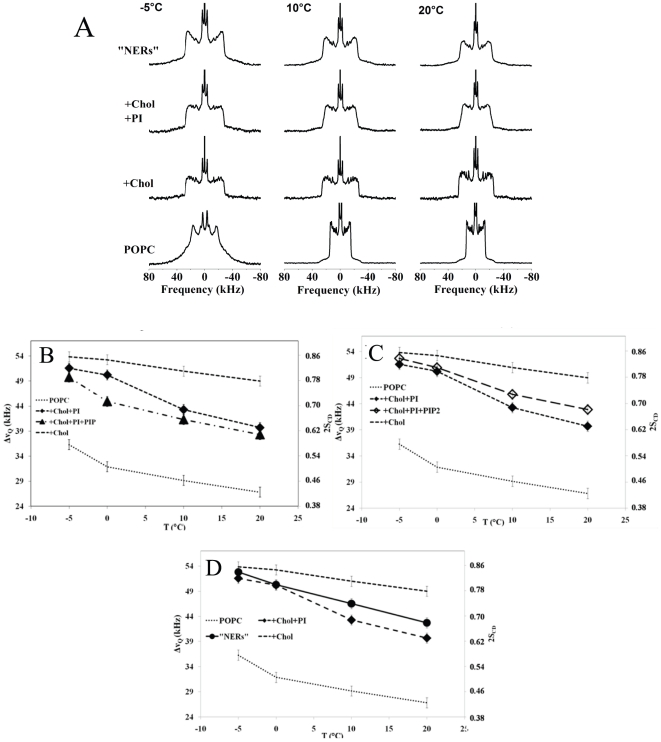
Core fluidity of PtdCho/Chol/PtdIns model membranes deuterium wide-line NMR spectra. **A**-Representative deuterium wide-line NMR spectra of POPC-^2^H with cholesterol in the absence or presence of different PtdIns. The molar ratios are representative of the lipid composition found in NER: POPC/Chol (58/42), POPC/Chol/PtdIns (28/42/30), and NERs-like membranes POPC/Chol/PtdIns/PtdIn**s**P/PtdInsP_2_ (28/42/23/16/7/7). Temperatures are indicated on the spectra. Sample hydration (water mass/water + lipid mass) is 95%. Depending on deuterated POPC amounts (1–3 mg), each spectrum is the result of 10 k to 100 k cumulative scans. A Lorentzian filtering (LB) of 200–300 Hz was applied prior to Fourier transformation. **FIG. 2B–D**. Ordering of chain segments close to glycerol backbone, NER-like membranes. Thermal variation of the *plateau* (k = 2 to 8–10) quadrupolar splittings of POPC/Chol/PtdIns (28/42/30) and POPC/Chol/PtdIns/PtdIn**s**P (28/42/23/7), panel B; POPC/Chol/PtdIns (28/42/30) and POPC/Chol/PtdIns/PtdIn**s**P_2_ (28/42/23/7), panel C: POPC/Chol/PtdIns (28/42/30) and NERs-like model membranes POPC/Chol/PtdIns/PtdIn**s**P/PtdInsP_2_ (28/42/23/16/7/7), panel D. For comparison, data for pure POPC and POPC/Chol (58/42) membranes was added to the graphs. Accuracy of the measure is ±1 kHz. On the double Y-axis the corresponding Carbon-Deuterium order parameter is shown. Because the average orientation of all *plateau* C–D bonds is at 90° with respect to the long lipid axis, twice |S_CD_| is plotted to express residual ordering information relative to the bilayer normal.

*In vivo* NER membranes differ from MV1 membranes in that they have elevated amounts of cholesterol. Figure 2A represents selected spectra of model membranes of increasingly complex composition (from bottom to top of the panel). Addition of cholesterol induced a liquid-ordered phase, *i.e.*, a decrease of order at low temperatures and an increase at high temperatures [29]. A sharp isotropic line (less than 5%) was detected, indicative of the formation of very small vesicles during sample preparation. In subsequent analyses this was negligible. In Figure 2A bottom row, the *so-ld* phase transition was not observed and the membrane rigidity was quasi-constant over a broad temperature range [28], [29], [30]. The addition of PtdIns to the POPC/Chol membrane system markedly decreased the width of cholesterol-containing POPC spectra. However, the original width of pure POPC spectra was not recovered and the signature of liquid ordered phases throughout the entire temperature range was still detected. Of note, a spectral broadening was observed, especially at high temperatures, suggesting intermediate exchange of POPC between distinct ordering environments [28]. PtdIns thus appeared to counterbalance the effect of cholesterol. A model membrane with a composition similar to NER (POPC/Chol/PtdIns/PtdInsP/PtdInsP_2_: 28/42/16/7/7 mole %) was measured (Figure 2A top row). The “NER-like” membranes had slightly wider spectra compared to POPC/Chol/PtdIns and also retained the signature of liquid ordered phase spectra (axially symmetric wide powder patterns over a large temperature range). The order parameter graphs (Figure 2B–D) illustrate the effects of PtdInsP_2_. The quantification of the order parameter was performed as in “MV1-like” membranes. Figure 2B shows the effect of cholesterol and PtdIns on POPC. As expected, cholesterol increased the order parameter of pure POPC over the entire temperature range. For example at 10°C, the addition of cholesterol to POPC increased the order parameter significantly from 0.47 to 0.81, indicating that the membrane tends towards a completely rigid and ordered system (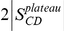 = 1). PtdIns slightly decreased the order parameter of the POPC/Chol membrane to 0.69, but compared to pure POPC the membrane was still ordered. Addition of PtdInsP (Figure 2B) decreased the order parameter compared to the POPC/Chol/PtdIns but at 10°C the variation was within experimental error. Conversely, addition of PtdInsP_2_ (Figure 2C) led to an increase in order especially at high temperatures. The order parameter properties of “NER-like” model membranes are shown in Figure 2D. Again the order parameter was greater than the POPC/Chol/PtdIns model but much less than the effect of cholesterol on POPC membranes alone. Therefore, membranes containing all three types of polyphosphoinositides were more fluid than those containing both POPC/Chol. However, they were still in a liquid-ordered phase, with an order parameter measured at the plateau positions of 0.73. For MV1 models, the deconvolution (de-Pakeing) of the spectra made it possible to measure accurately, the order parameters for chain positions, down to the terminal methyl group. A similar effect, as observed for the plateau positions, was detected for all chain segments (Figure S3).

In summary, polyphosphoinositides partially counterbalanced the effect of cholesterol by inducing molecular and intramolecular disorder at the hydrophobic core of the membrane.

### Structural dynamics at the membrane surface of MV1, MV2 and NER model membranes

**Figure 3:**
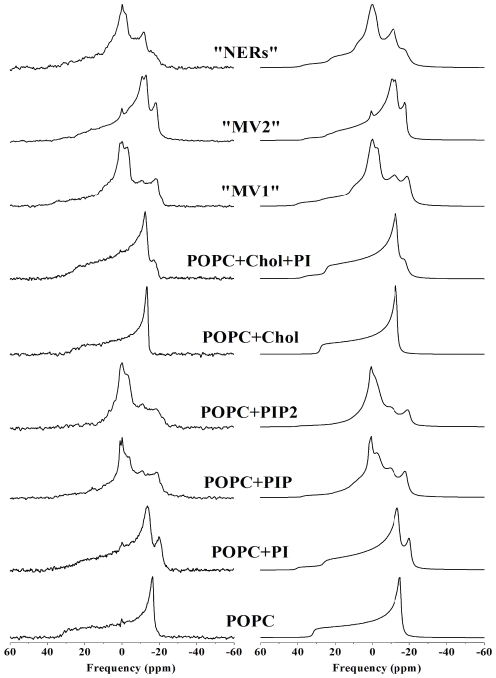
Orientational dynamics at the membrane surface determined by phosphorus-31 NMR spectra. Left Column: representative experimental wide-line phosphorus-31 NMR spectra of different model membranes containing POPC, PtdIns and cholesterol. The molar ratios are representative of the lipid composition found in MV1, MV2 and NERs, from bottoms to top: pure POPC, POPC/PtdIns (30/20), POPC/PtdInsP (30/18), POPC/PtdInsP_2_ (30/12), POPC/Chol (58/42), POPC/Chol/PtdIns (28/42/30), “MV1”: POPC/PtdIns/PtdInsP/PtdInsP_2_ (30/20/18/12), “MV2”: POPC/POPE/PtdIns/POPS (30/25/20/5) and NERs: POPC/Chol/PtdIns/PtdInsP/PtdInsP_2_ (28/42/23/16/7/7). Temperature is 10°C. Sample hydration (lipid mass/lipid + water mass) is 95%. Each spectrum is the result of 5 k cumulative scans. A Lorentzian filtering (LB) of 50–100 Hz was applied prior to Fourier transformation. Chemical shifts are expressed relative to 85% H_3_PO_4_ (0 ppm). Right column: simulated spectra according to procedures described in text. Initial guesses, as measured on de-Paked spectra, of chemical shielding anisotropies, Δσ, line widths, isotropic chemical shifts and relative weights of each subspectrum were supplied to the simulation procedure and iterative changes were performed until the best fit of experimental spectra was obtained. Δσ and isotropic chemical shifts are reported in Table 3.

Solid-state ^31^P-NMR enabled the comparison of the structural dynamics of the membrane surface in these model membranes. Figure 3 (left column) displays the experimental phosphorus NMR powder patterns for POPC, binary/ternary systems (POPC, individual polyphosphoinositides with and without cholesterol) and complex systems such as “MV1-like”, “MV2-like” and “NER-like” membranes at 10°C. We observed that spectra were all axially symmetric and contained up to seven powder patterns. PtdInsP_2_ on its own had three different Δσ, representing its phosphate groups (two phosphate groups on the inositol ring and one on the phosphodiester bond). The column on the right shows the calculated spectra. As described in the Materials and Methods, spectral simulations were performed by incorporating the appropriate values of Δσ, line width, isotropic chemical shielding and the molecular weight of each lipid component. In some cases a slight magnetic field deformation of MLVs was detected, indicated by line shapes initiating from the powder pattern spherical distribution of membrane orientations. This manifested by a decrease in the high frequency intensities detected on the left hand side of powder patterns. This prolate deformation was taken into account in the calculation, and the resulting Δσ are shown in Table 3.

**Table 3:** Chemical shielding anisotropies, Δσ, and isotropic chemical shifts δ_iso_ obtained from spectral simulations of experimental spectra of Figure 3.

	Δσ_P__C_	Δσ_P__E_	Δσ_P__S_	Δσ_P__I_	Δσ_P__I(4)P_	Δσ_P__I(4,5)P2_	Δσ_PI(4)P_	Δσ_PI(4,5)P2_	Δσ_PI(4,5)P2_
POPC	−47.0±1.5								
POPC/PI	−40.0±2.5			−61.0±2.5					
POPC/PIP	−34.5±2.5				−58.0±2.5		−14±2.5		
POPC/PIP2	−34.8±2.5					−60.0±4.0		−14±2.5	−6.0±2.5
“MV1-like”	−32.0±2.0			−60.0±5.0	−60.0±5.0	−60.0±5.0	−14±2.5	−14±2.5	−3.0±2.5
POPC/Chol	−40.8±1.5								
POPC/Chol/PI	−37.8±1.5			−54.9±1.5					
“NER-like”	−35.2±1.5			−56.0±3.5	−56.0±3.5	−56.0±3.5	−11.5±2.5	−11.5±2.5	−3.0±2.5
“MV2-like”	−38.0±1.5	−31.0±1.5	−51.0±1.5	−55.0±1.5					
δ_iso_ (ppm)	−1.0±0.5			−0.4±0.5	−0.3±0.5	−0.4±0.5	1.5±0.5	1.0±0.5	0.2±0.5

Initial estimates for Δσ and isotropic chemical shifts δ_iso_ were obtained from powder (non-oriented) or de-Paked (see text) spectra and were supplied to the simulation procedure together with estimates of line width and proportion of each phosphate according to sample composition. Calculated spectra were compared to experimental spectra and iterative changes were performed until the best fit was obtained. Accuracy is of 5–10% for large Δσ and up to 50% for smaller values. PI = PtdIns; PIP = PtdIn**s**P, PIP_2_ = PtdInsP_2_.

POPC chemical shift anisotropy decreased from ca. −47ppm to −40 ppm in the presence of PtdIns or cholesterol. It further decreased to ca. −35 ppm with PtdInsP or PtdInsP_2_, when added individually as well as for the “NER-like” membranes. For “MV1-like” membranes a further decrease to −32 ppm was observed. It was measured at ca. −38 ppm in POPC/Chol/PtdIns. The chemical shielding anisotropy of the phosphate bound to the glycerol backbone of phosphoinositides approached −60 ppm. However, for the additional phosphates in position (4,5) of the inositol ring, in the binary systems or “MV1-like” membranes, the values were much smaller, *i.e.*, −14 and −3 ppm, respectively. It is remarkable that the Δσ of the phosphoinositides in the binary mixtures or in “MV1-like” membranes remained whereas the Δσ of POPC decreased (Table 3).

This suggests that the phosphoinositides impose orientational dynamics on the other components at the membrane surface. For the corresponding phosphates of the “MV2- or “NER-like” membranes with cholesterol, smaller values of Δσ were measured.

The concept of phosphoinositides imposing orientational dynamics, on other components at the membrane surface, also holds true for “NER-like” and “MV2-like” model membranes but with a slight modulation induced by the presence of cholesterol. The variation in the magnitude of Δσ values in relation to intermolecular and molecular dynamics as well as the orientation of the phosphates group is discussed below.

## Discussion

Here we illustrate how polyphosphoinositides affect the core and membrane surface properties of nuclear membrane precursors in relation to nuclear membrane reassembly. The major finding is that the modulation of membrane fluidity is promoted by a mixture of PtdIns, PtdInsP and PtdInsP_2_ which are present in “MV1”, “MV2” and “NER”- like membranes. “MV1-like” membranes are unusually fluid whereas in the “NER-like” membranes, which contain elevated amounts of cholesterol, polyphosphoinositides reduce the ordering effect of cholesterol. Moreover, we indicate that phosphoinositides impose orientational dynamics on the other components at the membrane surface. The implication of these findings in relation to the physical properties of membranes and their possible biological role in membrane fusion and nuclear membrane assembly is discussed below.

### Polyphosphorylated phosphoinositides promote fluidity of nuclear envelope precursor membranes (“MV1-like” membranes)

Of all the phosphoinositides investigated, PtdIns induces the greatest effect on POPC bilayers; its disordering action is dose dependent and our results confirm those reported by [27]. PtdInsP and PtdInsP_2_ appear to mitigate the disordering effect of PtdIns. PtdInsP_2_ is particularly efficient because at low temperatures the initially observed *so-ld* phase transition reappears. However, the “MV1-like” membrane is as disordered as the simple POPC/PtdIns model. This indicates a very subtle interplay of ordering-disordering effects of the polyphosphorylated phosphoinositides when they are all present in the bilayer. It is also important to notice that spectra at 0°C and above exhibit a single average environment; this means that if domains of different fluidity were present they would exhibit a fast POPC exchange in the order of microseconds, which is within the deuterium solid-state NMR timescale. The chemical shielding anisotropy (Δσ) from the ^31^P NMR spectra provided information on the dynamics and average orientation of phosphate moieties with respect to the membrane normal [31], [32]. Addition of PtdIns to POPC membranes reduced Δσ. This reduction may be linked to a disordering effect as already observed in the case of the acyl chains. Addition of PtdInsP and PtdInsP_2_ led to a further decrease of Δσ. Because we showed that the phosphorylated phosphoinositides reversed the ordering effect of PtdIns, a further decrease in Δσ indicated a change in the orientation of the POPC phosphate. In order to obtain the accurate orientation from our data, an entire study using oriented samples would be required for determining the change in average orientation. These experiments are not within the scope of the current work.

For higher phosphorylated phosphoinositides (PtdInsP to PtdInsP_2_) the Δσ of the phosphate of the phosphodiester bond remained unchanged and was greater than the Δσ of POPC. This indicates different orientations for the glycerol-bound phosphates in PtdCho and in the phosphoinositides. This result is in agreement with a recent molecular dynamics study on membranes made of POPC and PtdInsP_2_ or PtdInsP_3_ where the axis of motional averaging was found to be perpendicular to the PO_3_ plane formed by the phosphorous atom and the two unlinked oxygen atoms [33] which are roughly within that plane in POPC [31]. The phosphoinositides appear to act as spacers of PtdCho, imposing a change in the phosphate orientation. PtdCho responds by changing its entire molecular dynamics and head group orientation. Chemical shielding anisotropy of the head group phosphates on the inositide ring is much smaller and is an ideal reporter of the orientation dynamics of the inositide moiety. The Δσ values of the 4-phosphate of PtdInsP and PtdInsP_2_ are within experimental error. This suggests that both orientation and dynamics of the inositol ring in PtdInsP and PtdInsP_2_ are the same. The Δσ of the 5-phosphate at is much smaller than the 4-phosphate of PtdInsP_2_ (Table 3). At the same temperature and hydration, the internal dynamics of the inositol ring can be considered to be constant, therefore indicating that 4- and 5-phosphates have different orientations with respect to the bilayer normal. The minute Δσ observed for position 5 suggests further that the z-axis of the phosphate tensor is close to 55° (an orientation at the magic angle, 54.7° would lead to Δσ = 0).

In complex “MV1-like” membranes all Δσ of phosphoinositides remain unchanged when compared to binary or ternary systems. This reinforces the idea that phosphoinositides are key players and without much modification of their own orientation dynamics they impose the orientation dynamics on other lipids. The imposition of orientation dynamics by the phosphoinositides may offer an explanation for the theoretical study of Pastor and co-workers [33], who suggested that clustering/electrostatic “bulging” of the inositide groups provides a more “visible” interaction for the recruitment of basic protein residues in the early events of signalling.

### Phosphoinositides counterbalance the cholesterol effect in nuclear envelope remnant model membranes

The major finding is that phosphoinositides reduce the ordering effect of cholesterol on the hydrophobic core of fluid membranes. The POPC model membrane containing both PtdIns and PtdInsP induces the greatest disordering effect. However, this effect is limited to a 20% decrease at 10°C, with the system still remaining in the liquid-ordered phase.

To our knowledge, this is the first class of phospholipids reported that is able to partially counterbalance the significant ordering effect of cholesterol. As observed for “MV1-like” model membranes, PtdInsP_2_ has a peculiar behaviour because it does not add to the disordering effect of PtdIns rather it acts in the opposite direction. The same is perceived for “NER-like” membranes where the total disordering promoted by the presence of the three phosphoinositide species is only reduced by 10% at 10°C. As already commented for “MV1-like” membranes, POPC spectra at 0°C and above report an averaged single environment. This assertion must however be toned down for the POPC/Chol/PtdIns system where spectral broadening may suggest an intermediate exchange between regions of different ordering within the microsecond time scale. The dynamics at the membrane surface is more subtle because complex interactions between cholesterol and phosphoinositide come into play.

As previously reported, cholesterol reduces the Δσ of POPC from −47 ppm to −40 ppm [32], [34]. This is known as the spacing effect, where cholesterol is well embedded in the membrane hydrophobic core with its -OH group facing the carbonyls of the acyl chains bound to glycerol [34]; the sterol fused ring systems condense the acyl chains and provide more space for the head groups. This translates to an increase in head group orientation dynamics, *i.e.*, an increase in intramolecular disorder and a change in the average orientation of the phosphate bound to the glycerol backbone. Δσs of all phosphoinositides are also reduced from ca. −60 ppm to ca. −55 ppm suggesting that cholesterol has a general effect on all lipids in the system, where it condenses the chains and liberates the head groups. Moreover, the concomitant effect of both cholesterol and phosphoinositides leads to a further decrease of the POPC Δσ.

The phosphates on the inositol ring have their Δσ slightly reduced compared to the Δσ of “MV1-like” membranes. This effect, as mentioned above, is probably linked to the general spacing effect of cholesterol.

### Biological implications for membrane fusion and nuclear envelope assembly

The involvement of the phosphoinositides and specifically PtdInsP_2_ and PtdInsP has been shown in vacuolar fusion in yeast, mammalian cells and nuclear envelope assembly [1], [2], [4], [35], [36], [37]. In vacuolar fusion it was suggested that they play a role in regrouping membrane SNAREs and their chaperones in domains so that the chaperones act synergistically with the SNAREs to induce fusion [36]. In the case of membrane fusion in nuclear envelope assembly, PtdInsP_2_ is modified to DAG by PLCγ, where fusion is initiated [7], [38]. In the third case where fusion at the plasma membrane was investigated, PtdInsP_2_ was proposed to be required for the recruitment of the SNARE fusion machinery [5], [6], [35]. Together, these examples show that higher phosphorylated phosphoinositides are involved in the mechanism of membrane fusion.

**Figure 4:**
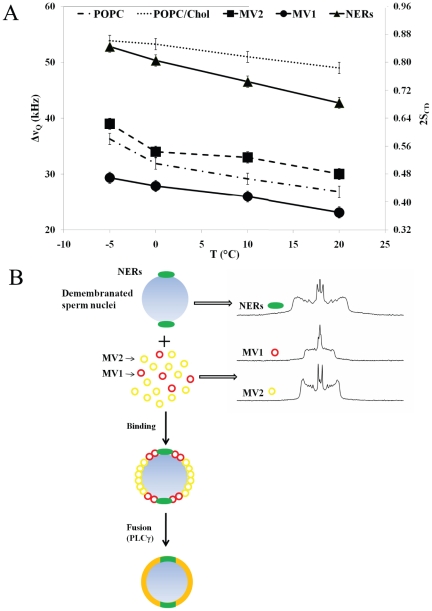
A- Distinct ordering of chain segments close to the glycerol backbone in MV1, MV2 and NERs like membranes. Thermal variation of the *plateau* (k = 2 to 8–10) quadrupolar splittings of MV1 MV2 and NERs model membranes. Data for POPC and POPC/Chol is also shown for comparison. Accuracy of the measure is ±1 kHz. On the double Y-axis the corresponding Carbon-Deuterium order parameter is shown. Because the average orientation of all *plateau* C–D bonds is at 90° with respect to the long lipid axis, twice |S_CD_| is plotted to express residual ordering information relative to the bilayer normal. FIG. 4B. Scheme describing how the physical properties of NER, MV1 and MV2 membranes could affect nuclear envelope assembly. Left: NERs (relatively-rigid) are located at the poles of the sperm nucleus and play the role of anchorage to chromatin. “MV1- like” membranes are very fluid and hence may have the role to “prime” the process of fusion. PLCγ in a second step hydrolyses PtdInsP_2_ into DAG, which initiates fusion of vesicles. Right: experimental deuterium NMR spectra of MV1, MV2 and NERs: the wider the trace the more rigid the system.

Our NMR studies offer a physical explanation of how the phosphoinositides may remodel membrane morphology to promote fusion. Figure 4A summarises the molecular ordering of the three model systems, “MV1, MV2 and NER-like” membranes. The data for POPC and POPC/Cholesterol have also been added to define the boundaries. It is clear that “MV1-like” membranes rich in phosphoinositides are unusually fluid (up to 20% increase compared to membranes with lower levels of phosphoinositides); NER membranes are from the liquid-ordered family but due to the presence of elevated amounts of phosphoinositides they are more disordered (10–15%) than PtdCho/Chol systems. “MV2-like” membranes have ordering properties that are between “MV1-like” and “NER–like” membranes.

Based on our findings we have drawn a scheme for *in vitro* nuclear envelope assembly, Figure 4B. The nuclear envelope remnants are membrane domains located at the acrosomal and centriolar fossae of the sperm nucleus. They are conserved membranous regions throughout the animal kingdom [39]. One of their physical properties is that they cannot be removed by moderate action of detergents. This may be related to the high amount of cholesterol (42 mol%) and to the manner in which they may anchor to chromatin [9].

As determined in this study these membranes, although having liquid-ordered phase properties, are nonetheless more fluid than classical liquid-ordered membranes. They may play a role as anchorage for chromosome reorganisation. In cell free assays MV1 has been demonstrated to primarily fuse to the poles of demembranated sperm nuclei at NERs [40]. Fusion propagates from the poles where MV1 is “docked” [40]. The unusual fluidity of these membranes would be ideal for the initial “docking” to more rigid NER membranes. From these studies we suggest that the initial “docking” of MV1 would be a priming event preparing the membrane environment for localised fusion to take place. Upon the activation of PLCγ on MV1, PtdIns(4,5)P_2_ is hydrolysed to DAG which induces localised fusion [7], [40]. Moreover, we have shown by ^31^P NMR studies that the polyphosphoinositides impose their orientation dynamics on other lipids. Under these circumstances the polyphosphoinositides could induce a clustering effect, which may enhance the “bulging” of a region of negative potential and hence the interaction with PH domains, C2 domains and polybasic rich residues of various proteins [33], [41], [42], [43], [44]. These types of interactions may counterbalance membranes with high amounts of phosphoinositides and render them stable.

The work reported here leads to a plausible explanation for the effect of polyphosphorylated phosphoinositides on membrane morphology, supporting also the theoretical work performed by Pastor and co-workers, and hones the concept that the polyphosphoinositides have a dual property of being signalling molecules as well as modulators of membrane morphology.

## Supporting Information

Figure S1 **Order Parameter of end chain segments C16-^2^H_3_, “MV1-like” membranes.** Thermal variation of the *C16-^2^H_3_ (k = 16) quadrupolar splittings of POPC*, POPC/PtdIns (30/20 and 10/40) panel A; POPC/PtdIns/PtdInsP (30/20/18), panel B; POPC/PtdIns/PtdInsP2 (30/20/12), panel C; and MV1-like model membranes POPC/PtdIns/PtdInsP/PtdInsP2 (30/20/18/12), panel D. For comparison, data for pure POPC and POPC/PtdIns (30/20) membranes were added to the graph for the three last compositions. Other parameters are as in main text.
(EPS)

Figure S2 **Core fluidity of “MV2-like” model membranes.** Representative deuterium wide-line NMR spectra of pure POPC-2H (left column) and with a MV2 composition: POPC/Chol/PtdIns/PtdEth/PtdSer (30/20/20/25/5). Temperatures are indicated on the spectra. Other parameters are as in main text.
(EPS)

Figure S3 **Order Parameter of end chain segments, “NER-like”-membranes.** Thermal variation of the *C16-^2^H_3_ (k = 16)* quadrupolar splittings of POPC/Chol/PtdIns (28/42/30) and POPC/Chol/PtdIns/PtdInsP (28/42/23/7), panel A; POPC/Chol/PtdIns (28/42/30) and POPC/Chol/PtdIns/PtdInsP2 (28/42/23/7), panel B: POPC/Chol/PtdIns (28/42/30) and NERs-like model membranes POPC/Chol/PtdIns/PtdInsP/PtdInsP2 (28/42/23/16/7/7), panel C. For comparison, data for pure POPC and POPC/Chol (58/42) membranes were added to the graphs. Other parameters are in main text.
(EPS)
